# A Burkitt's Lymphoma Case Mimicking Crohn's Disease: A Case Report

**DOI:** 10.1155/2011/685273

**Published:** 2011-09-08

**Authors:** Gulbanu Erkan, Mehmet Çoban, Aysun Çalıskan, Gokçe Kaan Ataç, Kamil Gulpınar, Bulent Degertekin, Atilla Korkmaz

**Affiliations:** ^1^. Department of Gastroenterology, Faculty of Medicine, Ufuk University Hospital, Mevlana Bulvarı No. 86-88, 06520 Balgat, Ankara, Turkey; ^2^Department of Radiology, Faculty of Medicine, Ufuk University Hospital, Mevlana Bulvarı No. 86-88, 06520 Balgat, Ankara, Turkey; ^3^Department of General Surgery, Faculty of Medicine, Ufuk University Hospital, Mevlana Bulvarı No. 86-88, 06520 Balgat, Ankara, Turkey

## Abstract

Lymphomas are solid tumors that arise from lymphoid tissue and present themselves as Hodgkin's or non-Hodgkin's lymphoma. Particularly gastrointestinal lymphomas can be clinically confused with other gastrointestinal tumors as well as with diffuse and inflammatory bowel disease. Early diagnosis and treatment bear vital importance in the management of lymphomas due to their high proliferation rates. In this report, we are presenting a case which initially displayed clinical and radiological signs of Crohn's disease, but was eventually diagnosed as Burkitt's lymphoma by laparotomy, and also we aim to underscore the importance of differential diagnosis.

## 1. Case Report

A 49-year-old male patient presented to our hospital because of abdominal pain, nausea-vomiting, lack of appetite, and weight loss (4 kg in the past month). He had been suffering abdominal pain in the past 3 weeks, which was localized in the right-lower quadrant, and it was relievable by defecation. During the past month, he had started to suffer diarrhea with no mucus and occasional appearance of fresh blood in the stool 2-3 times a day along with vomiting 1-2 times a day. His history revealed no remarkable event other than a hydatid cyst and simultaneous cholecystectomy operation 12 years previously. He had no history of immunosuppressive treatment. Physical examination revealed an incisional scar of 20 cm in length starting from the xiphoid process and extending parallel to the costal arch and towards the right axillary line, along with a mass lesion of 22 × 14 cm in size with an upper border 3 cm above the umbilicus and a lower border extending to the suprapubic region which was solid, deep, and painful upon palpation. 

CBC results were as follows: WBC: 8400/*μ*L; lymphocyte count: 1800/*μ*L; Hb: 10.20 g/dL; MCV: 84.1 fL; platelet: 527000/mm^3^. Biochemical profile was normal. Sedimentation rate was 60 mm/h, and CRP was 50 mg/L. There was no occult blood or parasite in the stool. Fecal culture showed no pathogenic bacterial growth. CEA, CA19-9, and CA 72-4 values were normal. The patient was seronegative for anti-HIV antibody and anti-EBV IgM and IgG. The patient was admitted to our hospital based on the presumptive diagnosis of Crohn's disease, lymphoma, colon cancer, or abscess.

**Figure 1:**
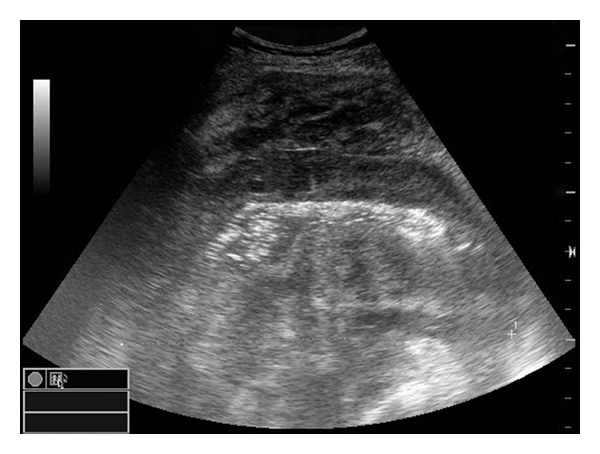
Ultrasonographic image displaying solid areas consistent with thickening of the intestinal wall in the right-lower quadrant.

Abdominal ultrasonography displayed calcifications located in the segment VI of the liver (postoperative changes?) and a 12 cm long intestinal segment of 5 cm total diameter and 1.6 cm thickness which was located superior to the bladder over the midline and left aspect of the midline (Figure 1). 

**Figure 2:**
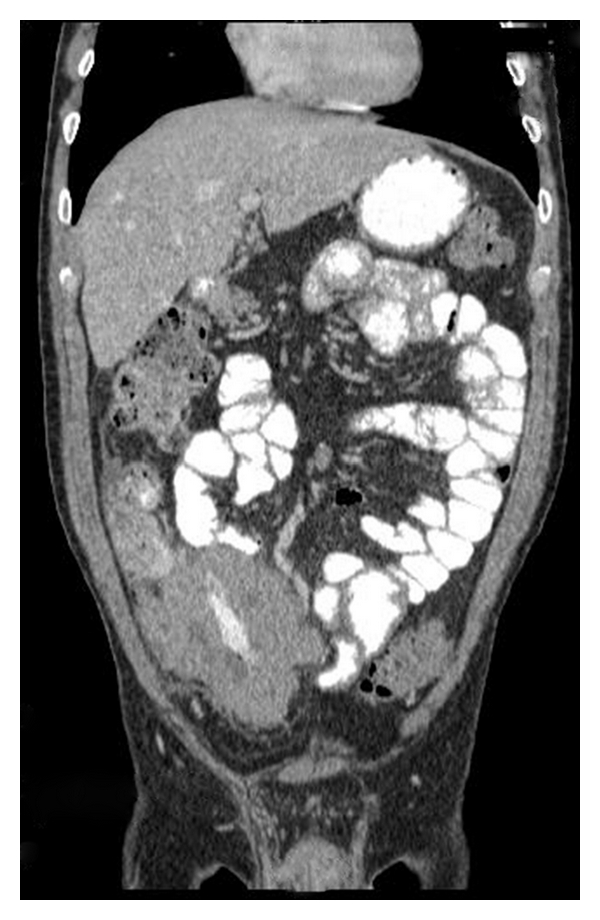
Coronal reformatted multislice abdominal computed tomography displaying a terminal ileum segment in the right-lower quadrant with marked thickening when compared to the adjacent ileal segments and showing mild enhancement after oral and intravenous contrast agent administration.

Abdominal tomography demonstrated coarse calcifications associated with the previous operation in the posterior aspect of the left liver lobe along with a heterogenous and solid area of 11 × 6 cm in size showing mild enhancement after intravenous contrast administration around which the adjacent adipose tissue had a streaky appearance (Figure 2). The center of the above-described lesion exhibited luminal enhancement in the intestines. Since the lumen of the small bowel was symmetrical, inflammation of the terminal ileum and its complications were considered first. Adenocarcinoma was ruled out based on the absence of luminal stenosis and lack of marked contrast enhancement.

The patient was subjected to total colonoscopy. Ileocecal valve was observed to be patent, and mucosa was found to be edematous and mildly irregular. Terminal ileal mucosa was severely edematous and granular. Due to the severe edema in the terminal ileum, the colonoscope could not be advanced further than 5 cm. There was no ulcer in the terminal ileum or ileocecal valve. Many biopsy samples were acquired from the ileocecal valve and terminal ileum. The lumen and mucosa of cecum, ascending colon, transverse colon, descending colon, sigmoid colon, and rectum were normal. Anal canal displayed internal hemorrhoids. 

Biopsies derived from ileum and ileocecal valve revealed chronic active nonspecific changes in two biopsy fragments and ulceration on the surface. Other fragments demonstrated edema and nonspecific inflammatory alterations. Although there was no granuloma, focal active inflammation was thought to be consistent with the Crohn's disease. 

**Figure 3:**
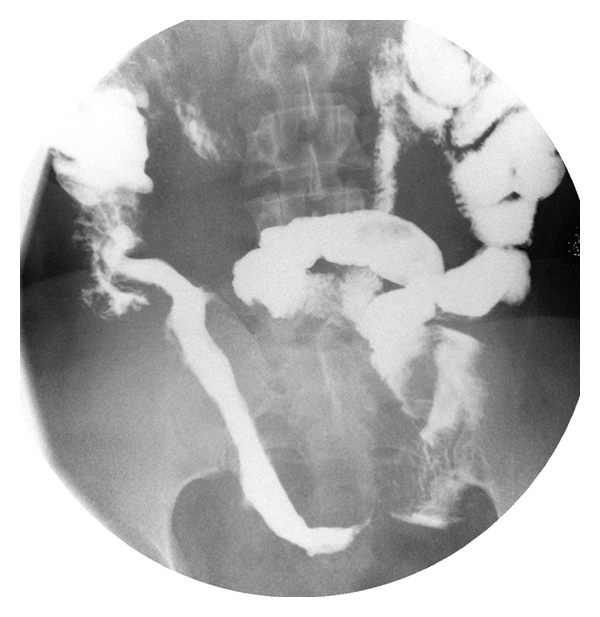
Barium small bowel passage radiogram displaying marked wall thickening involving a 40 cm long segment adjacent to the terminal ileum, characterized by displacement of the adjacent intestinal loops. No luminal irregularity, narrowing, or cavity formation was observed. These findings were interpreted as terminal ileal involvement of Crohn's disease.

Examination of the small bowel by barium contrast revealed an approximately 4 cm long segment that was extending proximally from cecum within the terminal ileum over which adjacent intestinal segments were pushed away, valvulae conniventes were indistinct, and small bowel mucosa had a patchy irregular appearance. Radiographic findings were thought to be consistent with Crohn's disease as well (Figure 3). 

**Figure 4:**
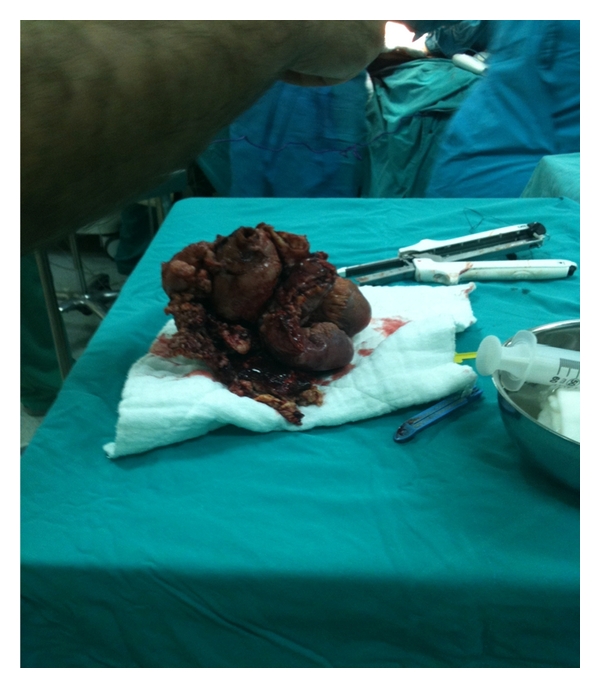
Macroscopic view of the mass resected from the terminal ileum after laparotomy.

Mesalazine therapy was started at a dose of 4 g/day. During the treatment of the patient, abdominal pain and diarrhea showed a regression; however, they recurred after 2-3 days of improvement. Inspections carried out on a daily basis revealed a remarkable and progressive increase in the size of the palpable mass over the right-lower quadrant. Lower extremity Doppler USG was performed due to diameter difference in favour of the right leg. Doppler USG depicted thrombosis in the right main femoral vein, deep femoral vein, proximal aspect of the superficial femoral vein, and proximal aspect of the great saphenous vein. A filter was placed into the vena cava inferior and low-molecular-weight heparin therapy was started. Because overall health status of the patient began to deteriorate progressively, he was transferred to surgery department for laparotomy. During the laparotomy, a tumoral lesion of approximately 20 cm in size was encountered in the ileum and resected (Figure 4). Histopathologic analysis of the resected material showed a malignant lymphoma consistent with CD20-positive atypical Burkitt's lymphoma.The patient died on the third postoperative day due to acute myocardial infarction and cardiac arrest which did not respond to cardiopulmonary resuscitation.

## 2. Discussion

Lymphomas are solid tumors of the lymphoid tissue. They are categorized in two groups as Hodgkin's lymphoma and non-Hodgkin's lymphoma. Hodgkin's lymphoma is rarely encountered in the gastrointestinal system. Non-Hodgkin's lymphomas generally arise from lymph nodes, and the lymphomas originating from other organs are termed as extranodal or primary lymphomas. More than 40% of extranodal lymphomas are seen in the gastrointestinal system, particularly in the stomach and small bowel. Majority of those are B-cell lymphomas. Most common ones are diffuse large cell lymphoma and marginal zone lymphoma of mucosa-associated lymphoid tissue (MALT). Other B-cell lymphomas are Burkitt's lymphoma, mantle cell lymphoma, and follicular lymphoma. 1-2% of adult non-Hodgkin's lymphomas are Burkitt's lymphoma. Burkitt's lymphoma is more frequently encountered during the childhood and in HIV-positive patients [1]. 

The association of Crohn's disease and non-Hodgkin's lymphoma is a contentious issue. Some immunologic changes in Crohn's disease are believed to create a predisposition for lymphoma development. Maclaurin et al. determined that lymphocyte reactivity against tumor cells was disrupted in patients with Crohn's disease [2]. Moreover, diseases associated with chronic inflammation, such as Hashimoto's thyroiditis, myoepithelial sialoadenitis, *Helicobacter*-related gastritis, rheumatoid arthritis, and Sjögren's syndrome, generate a predisposition for lymphoma development, as well [3]. Epstein-Barr virus (EBV) positivity increases in HIV-positive cases and patients undergoing immunosuppressive therapy. EBV reactivation and infection risk may be elevated in patients receiving immunosuppressive therapy. In cases where Crohn's disease and lymphoma coexist, EBV infection is encountered, as well [4, 5]. 

In the study of Lewis et al. which was conducted on 6605 Crohn's disease, 10.391 ulcerative colitis, and 60.506 control cases, lymphoma risk was not found to be elevated [6]. In another meta-analysis published later on by Kandiel et al., non-Hodgkin's lymphoma frequency was observed to be 4 times higher among inflammatory bowel disease patients receiving azathioprine and/or 6-mercaptopurine [7]. 

Moreover, there are cases of lymphoma mimicking Crohn's disease in the literature. Hurlstone reported an early-phase mantle cell lymphoma case macroscopically mimicking Crohn's disease in the terminal ileum [8]. McCullough et al. determined a mantle zone lymphoma mimicking diffuse inflammatory bowel disease which was diagnosed by immunohistochemical analysis [9]. 

There are similarities between small bowel lymphoma and Crohn's disease. In a study group, among radiological examinations of 50 patients diagnosed with Crohn's disease and/or lymphoma, 12 patients were found to have lymphoma associated with Crohn's disease, and differential diagnosis for Crohn's disease could not be established by radiography. Authors reported that stenosis, aneurysmal dilatation, ulceration, fistula formation, mesenteric mass, and other isolated or discontinued lesions may be present in the terminal ileum showing a nodular pattern and an inflammatory appearance [10]. 

Another characteristic feature of this patient was the complication of deep vein thrombosis. Lower extremity Doppler USG performed due to diameter difference in favour of the right leg revealed thrombosis in the femoral and saphenous veins. There is an elevated risk for thromboembolism in both lymphoma and inflammatory bowel disease [11, 12]. Mohren et al. performed a study on 1038 lymphoma cases and found thromboembolism in 7.7% of the patients [11]. Zhou et al. conducted a study on 422 patients and determined thromboembolism in 17.1% of them. The risk factors for thromboembolism in multivariate logistic regression analysis were noted to be female gender, elevated hemoglobin or creatinine levels, and chemotherapy with doxorubicin or methotrexate [13]. 

In our case, both tomography and small bowel radiograms showed findings mimicking Crohn's disease. However, the patient was subjected to urgent laparotomy due to presence of a palpable and rapidly growing mass lesion in the right-lower quadrant, rapid deterioration of the overall health status, and lack of clinical improvement despite mesalazine therapy. During the laparotomy, a tumoral mass of 20 cm in size was discovered and resected. Histopathologic examination results of the mass were consistent with Burkitt's lymphoma. 

Burkitt's lymphoma is generally encountered during the childhood period and in HIV-positive patients. Although it may present with symptoms reminiscent of Crohn's disease, it should be borne in mind that in cases that have a rapidly growing mass in the abdomen and fail to respond to mesalazine therapy, there is a likelihood of lymphoma presence which would require an explorative laparotomy for verification. Early diagnosis and treatment is of utmost importance because of the high proliferation rates in lymphomas. To the best of our knowledge, there is no Burkitt's lymphoma case mimicking Crohn's disease in the literature. Our case was deemed remarkable because of the presence of a Burkitt's lymphoma mimicking Crohn's disease which was complicated with thromboembolism, despite having no risk factors for lymphoma development such as HIV positivity or history of immunosuppressive drug use.
